# Supplementary material for: Enhanced activity of Alzheimer disease-associated variant of protein kinase Cα drives cognitive decline in a mouse model
Source: Nat Commun. 2022 Nov 23;13:7200. doi: 10.1038/s41467-022-34679-7 (PMC9684486; doi:10.1038/s41467-022-34679-7)
Supplement: Supplementary file 1 — Supplementary information [file 41467_2022_34679_MOESM1_ESM.pdf]

Enhanced Activity of Alzheimer Disease-associated Variant of PKCα Drives Cognitive Decline in a Mouse Model

Gema Lordén[1], Jacob M. Wozniak[1,2], Kim Doré[3], Lara E. Dozier[4], Chelsea Cates-Gatto[5], Gentry N. Patrick[4], David J. Gonzalez[1,2], Amanda J. Roberts[5], Rudolph E. Tanzi[6], Alexandra C. Newton[1*]

[1]Department of Pharmacology, University of California San Diego, La Jolla, CA 92093.

[2]Skaggs School of Pharmacy and Pharmaceutical Sciences, University of California San Diego, La Jolla, CA 92093.

[3]Center for Neural Circuits and Behavior, Department of Neurosciences, University of California San Diego, La Jolla, CA 92093.

[4]Section of Neurobiology. Division of Biological sciences, University of California San Diego, La Jolla, CA 92093.

[5]Animal Models Core Facility, The Scripps Research Institute, La Jolla, CA 92037.

[6]Genetics and Aging Research Unit, McCance Center for Brain Health, Department of Neurology, Massachusetts General Hospital and Harvard Medical School, Charlestown, MA 02129.

*Correspondence to: anewton@health.ucsd.edu

Supplementary information includes:

Material and Methods

Figures S1 to S4

Captions for Figures S1 to S4

Supplementary Tables T1, T2, T3

Captions for Tables T1 to T3

References

Uncropped scans of immunoblot from supplementary figures

Table dataset S1 – Submitted as a separated file

Table dataset S2 – Submitted as a separated file

The Mass spectrometry proteomics data were deposited to the MassIVE data base.

CONTACT FOR REAGENT AND RESOURCE SHARING

Further information and requests for resources and reagents should be directed to and will be fulfilled by the Lead Contact, Alexandra Newton (anewton@ucsd.edu).

SUPPLEMENTARY METHODS

Behavioral tests

Locomotor activity test. Locomotor activity was measured in polycarbonate cages (42 x 22 x 20 cm) placed into frames (25.5 x 47 cm) mounted with two levels of photocell beams at 2 and 7 cm above the bottom of the cage (San Diego Instruments, San Diego, CA). These two sets of beams allowed for the recording of both horizontal (locomotion) and vertical (rearing) behavior. A thin layer of bedding material was applied to the bottom of the cage. Mice were tested for 120 minutes and data were collected in 5-minute intervals.

Light/dark test. The light/dark transfer procedure has been used to assess anxiety-like behavior in mice by capitalizing on the conflict between exploration of a novel environment and the avoidance of a brightly lit open field [1]. The apparatus is a rectangular box made of Plexiglas divided by a partition into two environments. One compartment (14.5x27x26.5 cm) is dark (8-16 lux) and the other compartment (28.5x27x26.5 cm) is highly illuminated (400-600 lux) by a 60 W light source located above it. The compartments are connected by an opening (7.5x7.5 cm) located at floor level in the center of the partition. The time spent in the light compartment is used as a predictor of anxiety-like behavior, i.e., a greater amount of time in the light compartment is indicative of decreased anxiety-like behavior. Mice were placed in the dark compartment to start the 5-minute test.

ANOVA was used for the statistical analyses of behavioral results, followed by *post hoc* Student's *t*-tests as appropriate to determine the p values.

Kinase assay

Sample Preparation. Whole brain was lysed and homogenized as indicated in the main section of material and methods. Protein was quantified using a BCA protein assay kit (Thermo Fisher Scientific), and 2 mg of protein in 500 μl of RIPA buffer was incubated for 16 h at 4 °C with 2 μg of anti-PKCα (BD transduction laboratories, #610108) or its isotype control, IgG mouse (Santa Cruz, sc-2025). PKCα was

immunoprecipitated with 50 µl of Protein A/G beads (Santa Cruz, sc-2003) for one hour at 4 °C and used to perform a kinase assay. Purity of the immunoprecipitated protein was assessed by Coomassie staining.

Kinase Assay. The activity of immunoprecipitated PKCα WT or M489V toward a peptide substrate (Ac-FKKSFKL-NH2) was determined as described previously [2, 3]. Briefly, immunoprecipitated protein was resuspended in 48 µl of reaction buffer containing 50 mM Hepes, pH 7.5, 0.1 mg/mL BSA, and 2 mM DTT and then incubated in an 80 µl reaction volume containing 50 mM Hepes, pH 7.5, 100 µM ATP, 50 µCi ^{32}P-ATP (Perkin Elmer), 100 µM substrate, 5 mM $MgCl_2$, 0.06 mg/mL BSA, 1 mM DTT, and 500 µM EGTA without (non-activating conditions) or with (activating conditions) 700 µM $CaCl_2$ (corresponding to 200 µM free Ca^{2+}) and Triton X-100 (0.1% wt/vol) mixed micelles containing 15 mol % PS and 5 mol % DG. Following 5 min at 30 °C, reactions were quenched by addition of 25 mM ATP, 25 mM EDTA, pH 8; 5 µl were removed for immunoblot to verify that equal amounts of PKCα WT and M489V were employed in the kinase assay and 85 µl was prepared for analysis of ^{32}P-incorporation as described previously. [4]

PKCα-M489V Mouse

The C57BL/6NTac-Prkca mice containing the M489V mutation in *Prkca* were generated by Taconic Biosciences GmbH. Along with the point mutation, an AflIII restriction site (A/CRYGT) was introduced in the sequence to facilitate genotyping. To verify the successful insertion of the mutation in the genome, genomic DNA was isolated from mouse tails. The fragment containing the M489V mutation was amplified by PCR (Primer forward, 5'- GAGTGTCACTGAGACAAGCTAGG -3', and primer reverse, 5'-TGTCTCACTCAACATCTACAGGC-3') and subjected to Sanger sequencing. Our sequencing data indicates that the Met to Val was successfully inserted (Supp Figure 1A).

Amyloid-β analysis

ELISA: Brain tissues from APP transgenic mice harboring WT or mutant M489V PKCα were snap-frozen in liquid nitrogen and stored at -80 °C until use. Frozen brains were then homogenized in a Dounce tissue grinder and proteins sequentially extracted as previously described [5]. Aβ levels in 2% SDS and formic acid extracts were quantified using the Human/Rat Aβ-40 and Aβ-42 ELISA kits from Wako (#294-62501 and #292-64501 respectively), following the manufacturer's recommendations.

HISTOLOGY: Formalin-fixed brains were embedded in paraffin and cut into 4 μm sections. Deparaffinized sections were immuno-stained using a specific antibody for amyloid-β (clone 6E10, 1:400 dilution). For quantitative analysis of the Amyloid-β plaques in WT tg-AD and M489V tg-AD mice, cerebral cortex and hippocampus (n= four mice per group, approximately 4 fields per mice) of the brain were imaged using a Keyence BZ-9000E microscope. Quantifications were obtained by using the Image J v1.51k software to measure the total brain area analysis and the occupied area by amyloid-β. Percentage of amyloid-β area was determined by dividing the total area covered by amyloid-β by the total area in each brain region.

SUPPLEMENTARY FIGURES

Supplementary Figure 1

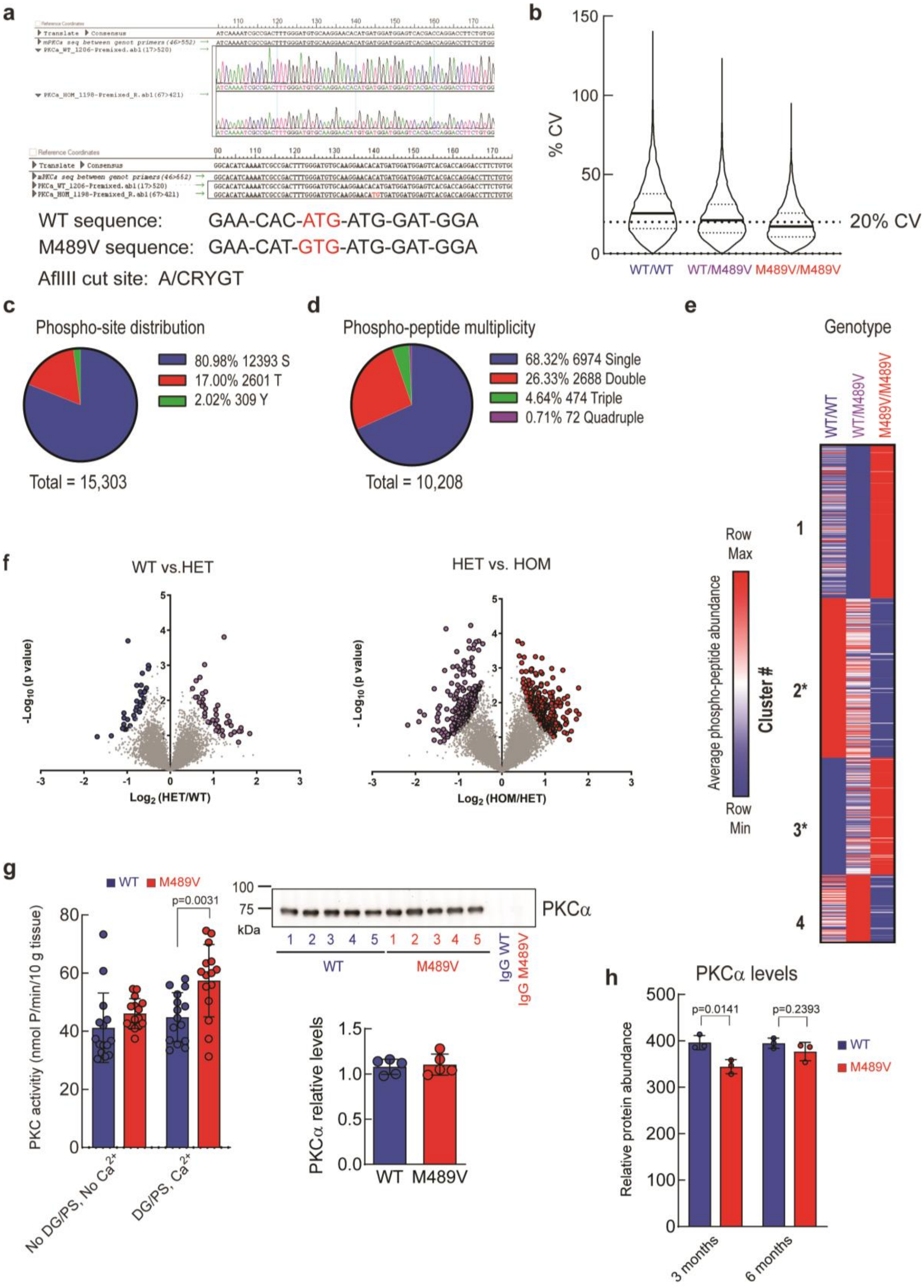

Supplementary Figure 1 (Supp to Fig 1): Phosphoproteomics analysis in brains from WT mice and mice harboring the PKCα M489V mutation on C57BL/6 background. A. Sanger-sequencing data from WT and M489V mice showing the correct insertion of the mutation. B. Co-efficient of variance plots for phosphoproteomics dataset 1 results. C. Pie charts of phosphosite distribution for the experiment in Figure 1. A total of 15,303 phosphosites were detected. D. Pie charts of phosphopeptide multiplicity for the experiment in Figure 1. A total of 10,208 peptides were quantified. E. Heatmap showing the distribution of significantly different peptides in the brains from different mice cohorts, highlighting the cluster 2 that contains peptides whose phosphorylation was decreased in a gene-dose dependent manner (WT/WT>WT/M489V>M489V/M489V), and cluster 3 that includes the peptides whose phosphorylation was increased in a gene-dose dependent manner (WT/WT<WT/M489V<M489V/M489V. Heatmap indicates whether the phosphorylation of a specific peptide is lower (blue) or higher (red). F. Volcano plot of phosphopeptides quantified from brains from WT mice vs. M489V heterozygous (HET) mice and HET vs. M489V homozygous (HOM) mice. The -log_{10}-transformed p-values associated with individual phosphopeptides are plotted against the log_2-transformed fold change in abundance between the compared groups. Significance was determined as in Figure 1B. G. Left. In vitro kinase assay showing the activity of PKCα WT (blue) or M489V (red) immunoprecipitated from murine brains. Data are graphed in units (nanomoles phosphate per minute) per 10 grams of tissue. Data represent the average ± SEM of samples (p values determined with unpaired two-tailed Student's *t*-test, n=5 biological independent samples). Uncropped blot included in this file. Right. Immunoblot of immunoprecipitated proteins (PKCα WT, M489V or IgG control) used in the kinase assay probed with antibodies for total PKCα. Graph shows the quantification of bands using densitometry and represents the average normalized intensity ± SEM. No statistical differences were found using a two-tailed Student's *t*-test. H. Graph showing the abundance of PKCα (average ± SD) in brains from WT (blue) and M489V homozygous (red) mice at 3 and 6 months of age (p values generated with a two-tailed Student's *t*-test, n=3 biological independent samples).

Supplementary Figure 2

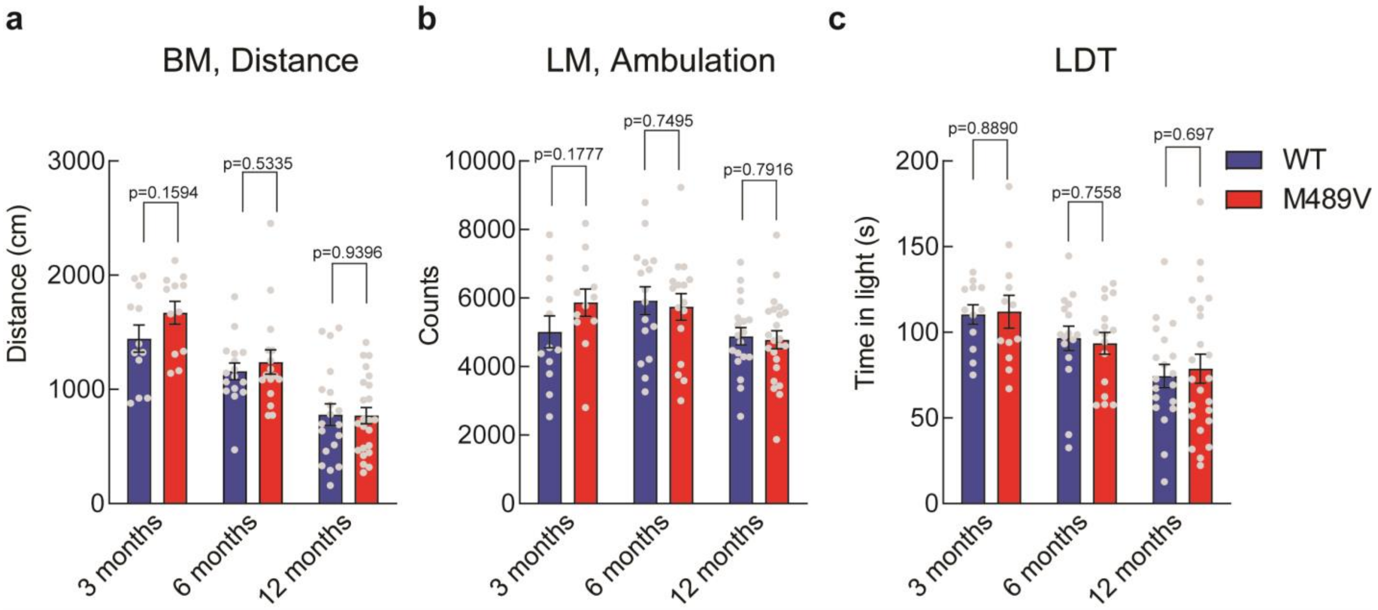

Supplementary Figure 2 (Supp to Fig 3): Additional behavioral tests on WT and M489V C57BL/6 mice. A. Distance traveled in the Barnes maze probe test (BM), B. ambulation in the locomotor test (LM), and C. time in light in the light/dark test (LTD) in PKCα WT and M489V mice at 3, 6 and 12 months mice. Same mice cohorts used in Figure 1 were subjected to these behavioral tests. Group sizes: 3 month (WT: 4 males, 8 females, M489V littermates: 6 males, 6 females), 6 month (WT: 8 males, 8 females, M489V littermates: 8 males, 8 females), 12 month (WT: 7 males, 12 females, M489V littermates: 12 males, 11 females). Error bars show standard error of the mean. ANOVA was used for statistical analysis and no statistical differences were found using a two-tailed Student's t-test. No sex effect was observed (n.s.= not significant). Source data are provided in the Source Data file.

Supplementary Figure 3

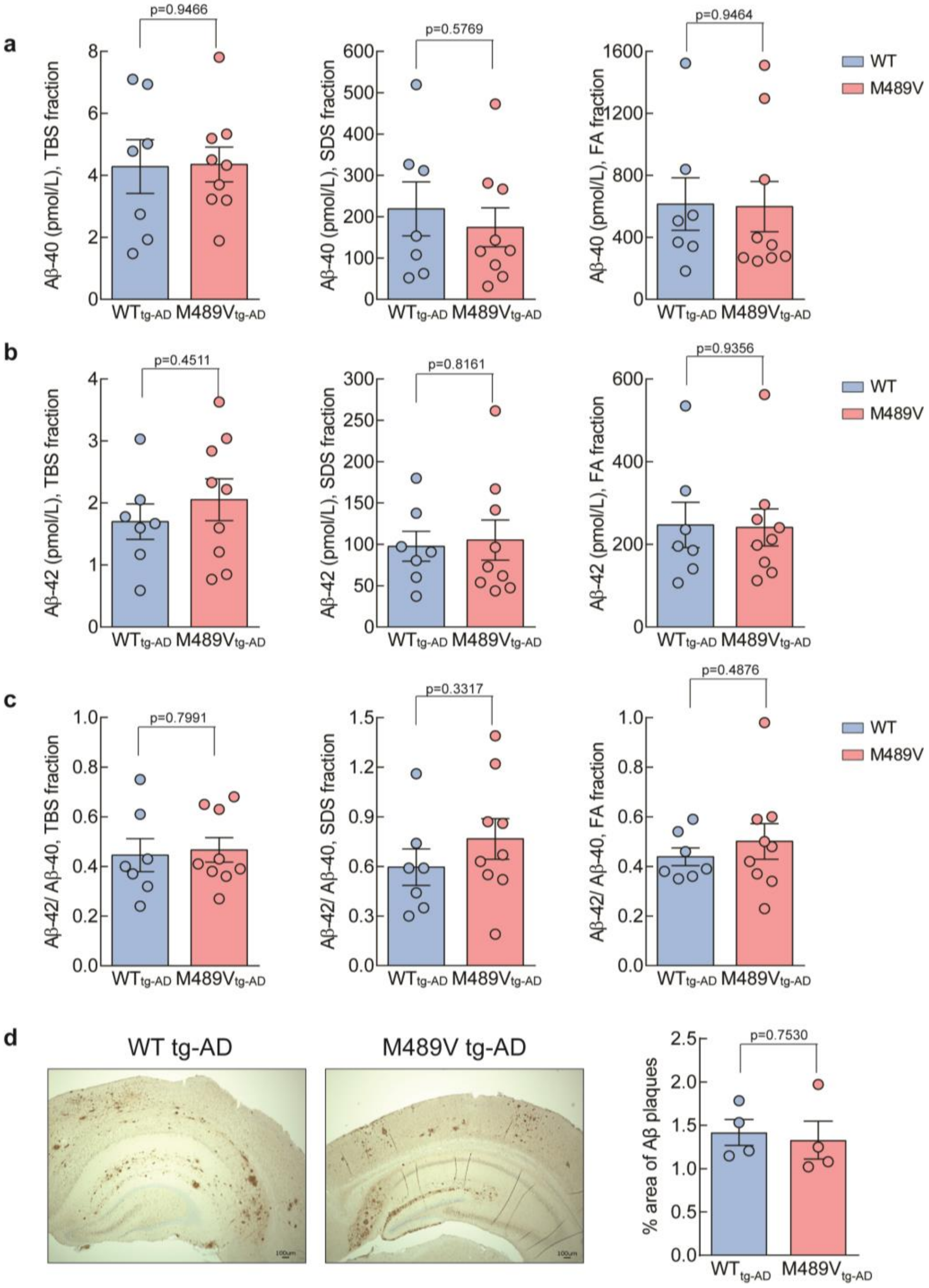

Supplementary Figure 3: Amyloid-β protein levels in brains from WT mice and mice harboring the PKCα M489V mutation on an AD transgenic mouse. Aβ-40 (A), and Aβ-42 (B) protein levels were quantified by ELISA in TBS soluble, SDS soluble and formic acid (FA) fractions of brain homogenates from WT tg-AD (blue) and M489V tg-AD (red) mice at 12 months of age. C. Aβ-42/ Aβ-40 ratios. The graphs in A, B, and C depict the average ± SEM. No significant differences between groups were found using a two-tailed Student's *t*-test (n=7 $WT_{tg\text{-}AD}$ and n=9 $M489V_{tg\text{-}AD}$ mice). D. Left, Representative image of Aβ staining by immunohistochemistry of WT tg-AD and M489V tg-AD mice brain. Scale bar represents 100 µm. Right. Quantification represents the average ± SEM of samples % area of Aβ in the brain. No statistical differences were found using a two-tailed Student's *t*-test (n=4 mice). Source data are provided in the Source Data file.

Supplementary Figure 4

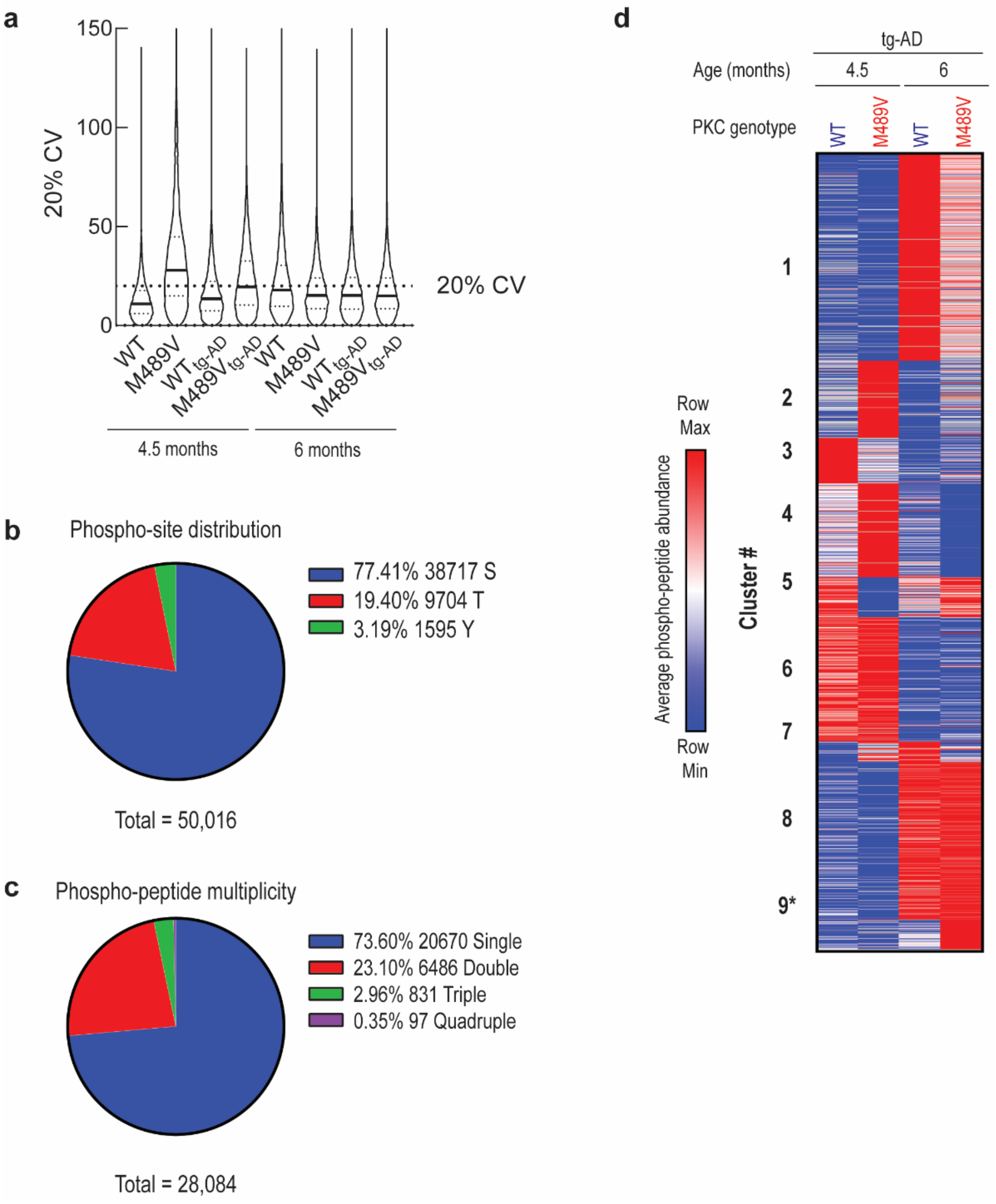

Supplementary Figure 4 (Supp to Fig 5): Phosphoproteomics analysis in brains from PKCα WT and M489V mice in the presence or absence of the APP transgene. A. Co-efficient of variance plots for phosphoproteomics dataset 2 results. B. Pie charts of phosphosite distribution for the experiment in Figure 5. A total of 50,016 phosphosites were detected. C. Pie charts of phosphopeptide multiplicity for the experiment in Figure 5. A total of 28,084 peptides were quantified. D. Heatmap showing the distribution of significantly different peptides in the brains from WT tg-AD and M489V tg-AD mice at 4.5 and 6 months, highlighting cluster 9 that contains peptides whose phosphorylation was increased in the M489V tg-AD mice 6 months old mice. Heatmap indicates whether the phosphorylation of a specific peptide is lower (blue) or higher (red). Source data for A, B, and C are provided in the Source Data file.

Supplementary Figure 5

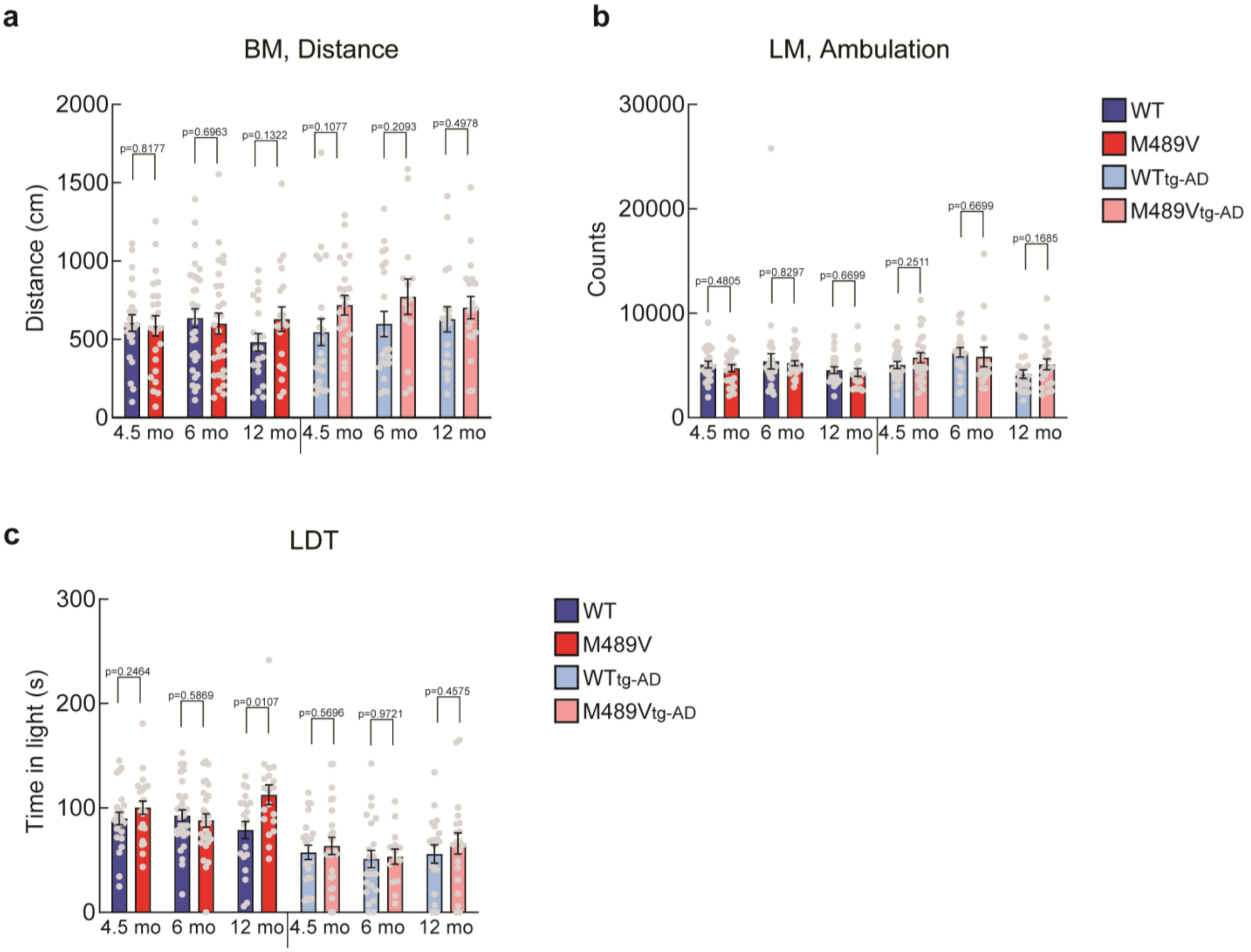

Supplementary Figure 5 (Supp to Fig 6): Additional behavioral tests on WT tg-AD and M489V tg-mice A. Distance traveled in the Barnes maze probe test (BM), B. ambulation in the locomotor test (LM), and C. time in light in the light/dark test (LDT) in WT non tg-AD (WT), M489V non tg-AD (M489V), WT tg-AD and M489V tg-AD mice at 4.5, 6, and 12 months. Group sizes: 4.5 month (WT non tg-AD (WT): 12 males, 12 females, M489V non tg-AD (M489V): 11 males, 12 females, WT harboring the APPswe transgene (WT tg-AD): 11 males, 11 females, M489V harboring the APPswe transgene (M489V tg-AD): 12 males, 12 females), 6 month (WT: 15 males, 16 females, M489V: 12 males, 16 females, WTAPP: 10 males, 11 females, M489VAPP: 8 males, 6 females), 12 month (WT: 10 males, 10 females, M489V: 10 males, 9 females, WTAPP: 9 males, 9 females, M489VAPP: 5 males, 14 females). Same mice cohorts used in Figure 6 were subjected to these behavioral tests. In A, B, and C error bars show standard error of the mean. ANOVA was used for statistical analysis followed by a two-tailed Student's *t*-test. ANOVA was used for statistical analysis, followed by post hoc two-tailed Student's t-test to determine the p values, and no statistical differences were found.No sex effect was observed. Source data are provided in the Source Data file.

SUPPLEMENTARY TABLES

Description	Annotated Sequence	ProtLoc	WT	M489V	FC	p value
[F-actin]-methionine sulfoxide oxidase MICAL3 =Mical3	sAGDQPPLLTPksPsDkELR	S1404.S1406	0.05	0.19	3.60	0.0122
Stromal membrane-associated protein 2 =Smap2	tSNALEkDLDLLASVPsPSSVSR	S221	0.06	0.20	3.42	0.0303
PC4 and SFRS1-interacting protein =Psip1	nLAkPGVTSTsDsEDEDDQEGEkkR	T269.S270.T271.S272.S274	0.06	0.18	3.08	0.0228
PC4 and SFRS1-interacting protein =Psip1	nLAkPGVTSTsDsEDEDDQEGEkkR	S272.S274	0.06	0.18	2.96	0.0179
PC4 and SFRS1-interacting protein =Psip1	nLAkPGVTSTsDsEDEDDQEGEkkR	S270.T271.S272.S274	0.06	0.18	2.95	0.0148
Stromal membrane-associated protein 2 =Smap2	tSNALEkDLDLLASVPsPSSVSR	S219	0.06	0.19	2.92	0.0270
Stromal membrane-associated protein 2 =Smap2	tSNALEkDLDLLASVPsPSSVSR	S219	0.07	0.19	2.72	0.0272
[F-actin]-methionine sulfoxide oxidase MICAL3 =Mical3	kSFVESVDEIPFADDVEDTYDDkTEDsSLQEk	T1529	0.06	0.17	2.71	0.0035
Stromal membrane-associated protein 2 =Smap2	tSNALEkDLDLLASVPSPSsVSR	S219	0.07	0.19	2.64	0.0454
Isoform Tau-A of Microtubule-associated protein tau =Mapt	iGStENLkHQPGGGk	T252	0.07	0.18	2.57	0.0228
NMDA receptor synaptonuclear signaling and neuronal migration factor =Nsmf	qHskLLDFDDVL	S523	0.07	0.18	2.51	0.0193
Sorting nexin-5 =Snx5	sVSVDLNVDPSLQIDIPDALSERDkVk	S20	0.08	0.20	2.49	0.0277
PC4 and SFRS1-interacting protein =Psip1	nLAkPGVTSTsDsEDEDDQEGEkkR	T271.S272.S274	0.07	0.17	2.45	0.0111
Microtubule-associated protein 2 =Map2	yTVPLPsPVQDSENLSGESGSFYEGTDDkVRR	S897	0.07	0.17	2.41	0.0402
Isoform 2 of Homer protein homolog 3 =Homer3	eksQDGGEFTSTGLALASHQVPPSPLVSTNGPGEEkLFR	S120	0.07	0.17	2.36	0.0208
ZW10 interactor =Zwint	nQsYLQLLcSLQNk	S216	0.08	0.18	2.34	0.0393
Cell division cycle 5-like protein =Cdc5l	skLVLPAPQISDAELQEVVk	S293	0.08	0.18	2.32	0.0243
Dynamin-1-like protein =Dnm1l	hLskGVEAEEWGk	S95	0.08	0.18	2.31	0.0332
Septin-7 =Sept7	iLEQQNSSRtLEkNk	S423	0.08	0.18	2.30	0.0065
PC4 and SFRS1-interacting protein =Psip1	nLAkPGVTStSDSEDEDDQEGEkkR	T271	0.07	0.17	2.30	0.0339
[F-actin]-methionine sulfoxide oxidase MICAL3 =Mical3	sAGDQPPLLTPksPSDkELR	S1404	0.08	0.18	2.29	0.0263
Epsin-3 =Epn3	dSAQALPTGksPSTVELDPFGDSSPSck	S445	0.09	0.20	2.29	0.0173
Synaptopodin =Synpo	hLEkVAsEEEEVPLVVYLk	S258	0.08	0.17	2.29	0.0243
Microtubule-associated protein 1A =Map1a	eMTLDQksPEkAk	T1813	0.06	0.15	2.27	0.0113
Brain-specific angiogenesis inhibitor 1 =Bai1	nENVATLSVSsLERR	S1467	0.07	0.17	2.27	0.0188

Supplementary Table 1: Top 25 phosphosites enhanced by the PKC M489V variant in C57 mice at 3 months old. Table shows the protein, phosphorylation sites and encompassing residues, WT and M489V values, fold change (FC) and the p value determined using a two-tailed Student's *t*-test not corrected for multiple hypotheses.

Description	Annotated sequence	ProtLoc	WT tg	M489V tg	FC	p value
Microtubule-associated protein =Mapt	AKTDHGAEIVYKSPVVSGDtsPR	T711.S712	0.18	0.46	2.51	0.03
Gap junction alpha-1 protein =Gja1	VAAGHELQPLAIVDQRPSSRASSRASSRPRPDDLEI_S18 S19 S23 S26_3	S364.S365.S369.S372	0.12	0.30	2.39	0.03
Electrogenic sodium bicarbonate cotransporter 1 =Slc4a4	NLTSSSLNDISDKPEKDQLK_S4 S5 S6_2	S255.S256.S257	0.16	0.31	1.97	0.01
Microtubule-associated protein =Mapt	AKTDHGAEIVYKSPVVSGDtSPR	T711	0.21	0.41	1.91	0.05
Isoform 2 of FACT complex subunit SSRP1 =Ssrp1	GLKEGINPGYDDYADsDEDQHDAYLER	S444	0.19	0.37	1.91	0.02
Amphiphysin =Amph	TPSPPEEPSPLPSPTASPNHTLAPASPAPVRPR_T1 S3 S17_2	T260.S262.S276	0.20	0.38	1.89	0.02
Protein piccolo =Pclo	SMsDPKPLSPTADESSRAPFQYSEGFTAK	S3610	0.17	0.32	1.87	0.02
Protein unc-80 homolog =Unc80	VASIQsEPGQQNVLLQQPLGR	S3113	0.21	0.36	1.73	0.05
Microtubule-associated protein =Mapt	AKTDHGAEIVYKSPVVSGDTSPR_T20 S21_1	T711.S712	0.22	0.37	1.68	0.05
Protein kinase C epsilon type =Prkce	SAPTSPCDQELKELENNIRK_T4 S5_1	T349.S350	0.23	0.38	1.65	0.00
Microtubule-associated protein =Mapt	AKTDHGAEIVYKSPVVSGDTsPR	S712	0.22	0.37	1.64	0.05
G protein-regulated inducer of neurite outgrowth 1 =Gprin1	KVDPPTTVEPVSLGKADsASPSPR	S618	0.20	0.32	1.64	0.05
Neuromodulin =Gap43	AEDGPAKEEPKQADVPAAVTDAAATtPAAEDAATK	T172	0.24	0.40	1.63	0.02
RalA-binding protein 1 =Ralbp1	TPSSEEISPTKFPGLYR_S3 S4 S8_2	S29.S30.S34	0.21	0.35	1.63	0.00
Electrogenic sodium bicarbonate cotransporter 1 =Slc4a4	ERISENYSDKsDVENADESSSSILKPLISPAAER	S68	0.19	0.30	1.57	0.04
Acidic leucine-rich nuclear phosphoprotein 32 family member A =Anp32a	NRtPSDVKELVLDNCK	T15	0.22	0.34	1.55	0.05
Cell cycle exit and neuronal differentiation protein 1 =Cend1	ADPVLLNNHSNLKPAPTVPAAPsSPDATSEPK	S89	0.24	0.38	1.54	0.05
Ubiquitin recognition factor in ER-associated degradation protein 1 =Ufd1	FIAFSGEGQsLRKK	S299	0.14	0.22	1.53	0.02
Disks large-associated protein 3 =Dlgap3	AIQAGCsQDDDCLPLLAAPASVSGRPGSSFNFRK	S510	0.17	0.26	1.53	0.01
Protein kinase C and casein kinase substrate in neurons protein 1 =Pacsin1	KAEGATLSNATGAVESTsQAGDR	S336	0.21	0.32	1.53	0.02
Caskin-1 =Caskin1	RKTPQSLEMMAIESPPPSEPAAAECQsPK	S650	0.21	0.31	1.51	0.03
Caskin-1 =Caskin1	sQEYLLDEGMAPGTPPK	S728	0.11	0.17	1.50	0.02
Paralemmin-1 =Palm	EIDVLEFGESAPAASKENSAAPsPGRPQSASPAKEEQK	S116	0.23	0.35	1.50	0.02
Inositol hexakisphosphate and diphosphoinositol-pentakisphosphate kinase 2 =Ppip5k2	RFFHHAEEEEEEEDEsPPER	S38	0.18	0.27	1.49	0.04
Leucine zipper protein 1 =Luzp1	EKPDsDDDLDIESFVTAK	S660	0.16	0.24	1.48	0.04

Supplementary Table 2: Top 25 phosphosites enhanced by the PKC M489V variant in tg-AD mice at 4.5 months old. Table shows the protein, phosphorylation sites and encompassing residues, WT and M489V values, fold change (FC) and the p value determined using a two-tailed Student's *t*-test not corrected for multiple hypotheses.

.

Description	Identifier	ProtLoc	WT tg	M489V tg	FC	p value
PH and SEC7 domain-containing protein 3 =Psd3	sHSSPSLNPDASPVTAK	S337	0.28	0.55	2.00	0.03
Calcium/calmodulin-dependent protein kinase type II subunit alpha =Camk2a	KNDGVKEsSESTNTTIEDEDTK	S330	0.14	0.25	1.73	0.02
DnaJ homolog subfamily C member 5 =Dnajc5	QRSLSTSGESLYHVLGLDK_S5 T6_1	S10.T11	0.19	0.31	1.66	0.03
Forkhead box protein K1 =Foxk1	SLVSPIPsPTGTISVPNSCPAsPR	S229.S243	0.17	0.28	1.64	0.03
Microtubule-associated protein 1B =Map1b	SDIsPLtPREsSPLYSPGFSDSTSAAK	S1781.T1784.S1788	0.18	0.30	1.63	0.05
Isoform 2 of Ankyrin-2 =Ank2	YVsSDGTEKEEVTMQGMPQEPVNIEDGDNYSK	S3878	0.20	0.32	1.62	0.01
Neurofilament 3, medium =Nefm	KGEDsSDDKVVVTK	S779	0.16	0.25	1.61	0.04
Nectin-1 =Nectin1	KAGPLGGSsYEEEEEEEGGGGGER	S434	0.15	0.25	1.59	0.04
AP-3 complex subunit beta-1 =Ap3b1	NFyESEEEEEEKEK	Y274	0.14	0.22	1.59	0.01
AP-3 complex subunit beta-1 =Ap3b1	NFYEsEEEEEEKEK	S276	0.14	0.22	1.59	0.04
GTP-binding protein 1 =Gtpbp1	LHGGFDSDCSEDGEALNGEPELDLTSK_S7 S10_1	S44.S47	0.14	0.22	1.57	0.03
Microtubule-associated protein =Mapt	SPVVsGDTsPR	S708.S712	0.09	0.15	1.56	0.02
PC4 and SFRS1-interacting protein =Psip1	QSNASsDVEVEEKETNVSKEDTDQEEK	S106	0.15	0.23	1.56	0.02
Anaphase-promoting complex subunit 4 =Anapc4	IKEEVLsESETEAHQDAAALDPDVVIK	S777	0.18	0.27	1.56	0.05
Slc24a2 protein =Slc24a2	IELPNSTSTEVEMTPSSEASEPVQNGNLSHNIEAADAPK_S6 T7 S8 T9_1	S463.T464.S465.T466	0.19	0.29	1.54	0.03
Protein PRRC2A =Prrc2a	LKFsDEEDGRDsDEEGAEGHK	S342.S350	0.14	0.22	1.53	0.01
Calcium/calmodulin-dependent protein kinase type II subunit alpha =Camk2a	ESSESTNTtIEDEDTKVR	T337	0.15	0.23	1.49	0.00
Plasma membrane calcium-transporting ATPase 1 =Atp2b1	AQDGAAMEMQPLKsEEGGDGDEK	S338	0.17	0.25	1.47	0.01
UPF0606 protein KIAA1549 =Kiaa1549	GHYEFPVVDDLssGDTK	S1543.S1544	0.21	0.31	1.46	0.05
Histone deacetylase =Hdac2	RIACDEEFsDsEDEGEGGRR	S422.S424	0.16	0.23	1.45	0.01
Hepatocyte cell adhesion molecule =Hepacam	SEADTLPRsGEQER	S301	0.15	0.22	1.44	0.02
Brain acid soluble protein 1 =Basp1	KAEGAGtEEEGtPKESEPQAAADATEVK	T31.T36	0.20	0.28	1.44	0.02
Protein kinase C and casein kinase substrate in neurons protein 1 =Pacsin1	GQTYATEWsDDESGNPFGGNEANGGANPFEDDAKGVR	S358	0.27	0.38	1.43	0.05
Serine/threonine-protein kinase PAK 1 =Pak1	SVIEPLPVTPTRDVATSPISPTENNTTPPDALTR_S17 S20 T22_2	S219.S222.T224	0.26	0.37	1.42	0.04
RIKEN cDNA 2010300C02 gene =2010300C02Rik	KIMAPPERDMsPSEGDVAPPK	S570	0.13	0.18	1.42	0.00

Supplementary Table 3: Top 25 phosphosites enhanced by the PKC M489V variant in tg-AD mice at 6 months old. Table shows the protein, phosphorylation sites and encompassing residues, WT and M489V values, fold change (FC) and the p value determined using a two-tailed Student's *t*-test not corrected for multiple hypotheses.

SUPPLEMENTARY REFERENCES

1. Crawley JN. Behavioral phenotyping of transgenic and knockout mice: experimental design and evaluation of general health, sensory functions, motor abilities, and specific behavioral tests. *Brain research* **835**, 18-26 (1999).

2. Callender JA*, et al.* Protein kinase Calpha gain-of-function variant in Alzheimer's disease displays enhanced catalysis by a mechanism that evades down-regulation. *Proc Natl Acad Sci U S A* **115**, E5497-E5505 (2018).

3. Keranen LM, Newton AC. Ca2+ differentially regulates conventional protein kinase Cs' membrane interaction and activation. *J Biol Chem* **272**, 25959-25967 (1997).

4. Tobias IS*, et al.* Protein kinase Czeta exhibits constitutive phosphorylation and phosphatidylinositol-3,4,5-triphosphate-independent regulation. *Biochem J* **473**, 509-523 (2016).

5. Kawarabayashi T, Younkin LH, Saido TC, Shoji M, Ashe KH, Younkin SG. Age-dependent changes in brain, CSF, and plasma amyloid (beta) protein in the Tg2576 transgenic mouse model of Alzheimer's disease. *J Neurosci* **21**, 372-381 (2001).

UNCROPPED SCANS OF IMMUNOBLOT FROM SUPPLEMENTARY FIGURES

For Supplementary Figure 1G

Epi white for PKCα

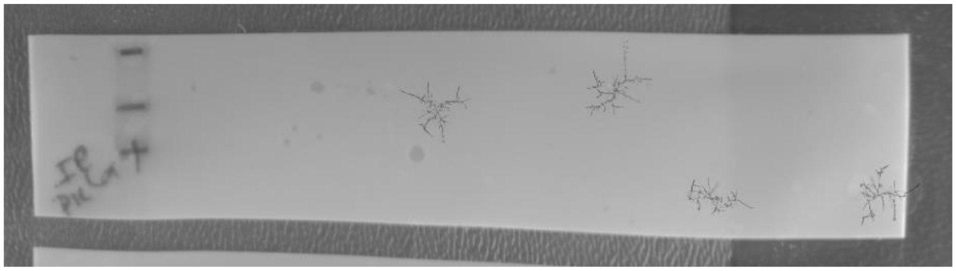

PKCα gel

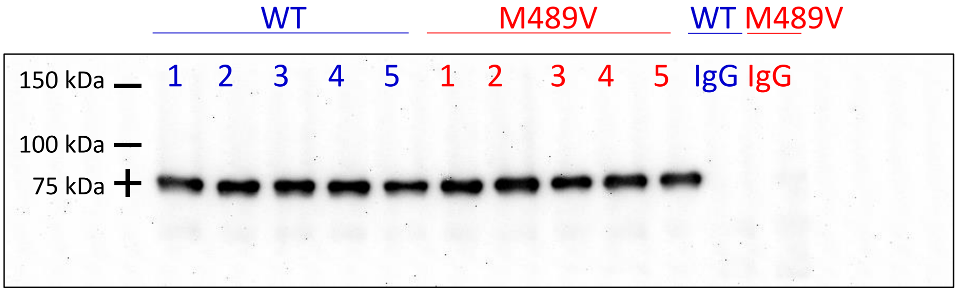

PKCα
