# Supplementary material for: Enhanced activity of Alzheimer disease-associated variant of protein kinase Cα drives cognitive decline in a mouse model
Source: Nat Commun. 2022 Nov 23;13:7200. doi: 10.1038/s41467-022-34679-7 (PMC9684486; doi:10.1038/s41467-022-34679-7)
Supplement: Supplementary file 3 — Description of Additional Supplementary Files [file 41467_2022_34679_MOESM3_ESM.pdf]

Description of Additional Supplementary Files

File Name: Supplementary Data 1

Description: Phosphoproteomics and proteomics data for analysis of brains from 3-month old WT mice and mice harboring the PKCα M489V mutation on C57BL/6 background.

File Name: Supplementary Data 2

Description: Phosphoproteomics and proteomics data for analysis of brains from WT mice and mice harboring the PKCα M489V mutation on a B6;SJL background with the APP transgene carrying the Swedish mutation (APPswe).
