# Supplementary material for: Enhanced activity of Alzheimer disease-associated variant of protein kinase Cα drives cognitive decline in a mouse model
Source: Nat Commun. 2022 Nov 23;13:7200. doi: 10.1038/s41467-022-34679-7 (PMC9684486; doi:10.1038/s41467-022-34679-7)
Supplement: Supplementary file 6 — Reporting Summary [file 41467_2022_34679_MOESM6_ESM.pdf]

nature portfolio

Corresponding author(s): Alexandra C. Newton

Last updated by author(s): Oct 28, 2022

Reporting Summary

Nature Portfolio wishes to improve the reproducibility of the work that we publish. This form provides structure for consistency and transparency in reporting. For further information on Nature Portfolio policies, see our Editorial Policies and the Editorial Policy Checklist.

Statistics

For all statistical analyses, confirm that the following items are present in the figure legend, table legend, main text, or Methods section.

n/a	Confirmed	
[ ]	[x]	The exact sample size (*n*) for each experimental group/condition, given as a discrete number and unit of measurement
[ ]	[x]	A statement on whether measurements were taken from distinct samples or whether the same sample was measured repeatedly
[ ]	[x]	The statistical test(s) used AND whether they are one- or two-sided<br>*Only common tests should be described solely by name; describe more complex techniques in the Methods section.*
[x]	[ ]	A description of all covariates tested
[x]	[ ]	A description of any assumptions or corrections, such as tests of normality and adjustment for multiple comparisons
[ ]	[x]	A full description of the statistical parameters including central tendency (e.g. means) or other basic estimates (e.g. regression coefficient) AND variation (e.g. standard deviation) or associated estimates of uncertainty (e.g. confidence intervals)
[ ]	[x]	For null hypothesis testing, the test statistic (e.g. F, t, r) with confidence intervals, effect sizes, degrees of freedom and P value noted<br>*Give P values as exact values whenever suitable.*
[x]	[ ]	For Bayesian analysis, information on the choice of priors and Markov chain Monte Carlo settings
[x]	[ ]	For hierarchical and complex designs, identification of the appropriate level for tests and full reporting of outcomes
[x]	[ ]	Estimates of effect sizes (e.g. Cohen's *d*, Pearson's *r*), indicating how they were calculated

Our web collection on statistics for biologists contains articles on many of the points above.

Software and code

Policy information about availability of computer code

Data collection	Commercial softwares were used for data collection.
Data analysis	Igor Pro 4.04 software, Proteome Discoverer 2.1, PTMphinder R package, Image J v1.51k, Image J v1.41o, Volocity v6.1.1, AlphaView SA (v3.4.0), and GraphPad Prism 9 were used for data analysis.

For manuscripts utilizing custom algorithms or software that are central to the research but not yet described in published literature, software must be made available to editors and reviewers. We strongly encourage code deposition in a community repository (e.g. GitHub). See the Nature Portfolio guidelines for submitting code & software for further information.

Data

Policy information about availability of data

All manuscripts must include a data availability statement. This statement should provide the following information, where applicable:

- Accession codes, unique identifiers, or web links for publicly available datasets
- A description of any restrictions on data availability
- For clinical datasets or third party data, please ensure that the statement adheres to our policy

The MS proteomics data generated for this manuscript, including annotated spectra, have been deposited onto the ProteomeXchange archive through MassIVE under the following identifiers: PKC-M489V 3 months old mice, C57BL/6N background (ProteomeXchange: PXD029092, https://doi.org/doi:10.25345/C59569), PKC-M489V 4.5 and 6 months old mice, APP transgenic background (ProteomeXchange: PXD029093, https://doi.org/doi:10.25345/C55C4C).
Uniprot human protein database was employed to query MS2 spectra (downloaded: 05/2017, https://www.uniprot.org/). The Database for Annotation, Visualization and Integrated Discovery (DAVID) was employed for gene ontology analyses (https://david.ncifcrf.gov/). The STRING database (v11.0) was used to predict protein-protein interactions (https://string-db.org/cgi/input?sessionId=bNKueBJyx4dL&input_page_show_search=off).

Field-specific reporting

Please select the one below that is the best fit for your research. If you are not sure, read the appropriate sections before making your selection.

☒ Life sciences ☐ Behavioural & social sciences ☐ Ecological, evolutionary & environmental sciences

For a reference copy of the document with all sections, see nature.com/documents/nr-reporting-summary-flat.pdf

Life sciences study design

All studies must disclose on these points even when the disclosure is negative.

Sample size	For behavioral studies, group sizes of at least 12 were based on power analysis and calculated from murine data collected in the laboratory of Dr. Roberts over the past 25 years (at least 32 papers directly using learning and memory tests, for example https://pubmed.ncbi.nlm.nih.gov/28431906/, https://pubmed.ncbi.nlm.nih.gov/32534984/). This group size is associated with powers of 92-97% for the behavioral tests employed in this study using the online calculator (http://powerandsamplesize.com/Calculators/).<br>For other experiments, sample size per group was determined from previous publications with similar methodologies. For example, for phosphoproteomic analyses, samples size were determined based in these two papers in which they used n=3 for some of their brain analysis (https://www.nature.com/articles/s41467-018-06519-0 and https://doi.org/10.1038/nn.4160).
Data exclusions	For the electrophysiology experiments, cells that had series resistance higher than 30 MOhms or holding current higher than 1pA were excluded. For the behavioral experiments, no data were excluded unless a mouse was either found dead or was deemed sick by Animal Models Core personnel (and confirmed by Scripps Research Animal Health Technicians) and was euthanized. In these cases, all previous data from these mice were removed from the analyses.
Replication	For behavioral studies, all mice for a specific experiment (i.e. set of genotypes at a particular age) were produced by Taconic and then shipped as large independent groups, making replication impossible. However, we tested these separate groups of mice at different ages and saw replication of certain phenotypes and age-related trends.<br>Phosphoproteomics analysis, three biologically independent samples for each genotype were analyzed in the same experiment (n=3). We did not repeat phosphoproteomic experiments with same cohort of mice, but hits were validated by western blot.<br>Western blot analysis were repeated independently at least three times with similar results.<br>For spine density analysis, n=6 mice were analyzed independently. Neurons from 1 mouse of each genotype were analyze each time. 5 out of 6 experiments showed decreased spine density in M489V mice compared to WT. 1 out of 6, did not show spine density reduction in M489V mice. All replicates used were included in the final analysis.
Randomization	The groups were based on genetics and not on any treatment or condition that we had control over. However, we did test the mice completely blind to genotype such that this factor was randomized throughout the process of behavioral testing.
Blinding	Investigators were blinded to genotype and for data analysis.

Reporting for specific materials, systems and methods

We require information from authors about some types of materials, experimental systems and methods used in many studies. Here, indicate whether each material, system or method listed is relevant to your study. If you are not sure if a list item applies to your research, read the appropriate section before selecting a response.

Materials & experimental systems

n/a	Involved in the study
☐	☒ Antibodies
☒	☐ Eukaryotic cell lines
☒	☐ Palaeontology and archaeology
☐	☒ Animals and other organisms
☒	☐ Human research participants
☒	☐ Clinical data
☒	☐ Dual use research of concern

Methods

n/a	Involved in the study
☒	☐ ChIP-seq
☒	☐ Flow cytometry
☒	☐ MRI-based neuroimaging

Antibodies

Antibodies used	anti-PKCα antibody was from BD Transduction Laboratories (610108, clone 3/PKCα, used at 1:1,000 dilution).<br>β-Actin antibody was purchased from Sigma-Aldrich (A2228, clone AC-74, used at 1:20,000 dilution).<br>total SAP97 was from Enzo Life Sciences (ADI-VAM-PS005, clone RPI 197.4, used at 1:1,000 dilution).<br>phospho-MARCKS (sc-12971-R) and total MARCKS (sc-6454) were obtained from Santa Cruz Biotechnology. Both were used at a 1:250 dilution.<br>GAPDH (2118, clone 14C10), vinculin (4650), phospho-(Ser) PKC substrate (2261S), phospho-ERK1/2 (9101) and total ERK1/2 (9102) antibodies were purchased from Cell Signaling Technology. All of them were used at a 1:250 dilution.

	The pSAP97 (T656) antibody was custom made by NeoMPS by immunizing rabbits with an Ac-CKERARLK-T(PO3H2)-VKFN-NH2 peptide that was conjugated to keyhole limpet hemocyanin and was affinity-purified using the phosphopeptide antigen, as described and characterized in O'Neill et al., 2010 (see below). It was used at a 1:1,000 dilution.<br>Amyloid-beta antibody (clone 6E10, used at a 1:400 dilution).
Validation	Antibodies were purchased from commercial sources. When possible, commercial antibodies with specific publications of previous use were purchased.<br>Validation on manufacturer's website or previous publication.<br>anti-PKCα (610108):https://www.bdbiosciences.com/en-us/products/reagents/microscopy-imaging-reagents/immunofluorescence-reagents/purified-mouse-anti-pkc.610108<br>β-Actin (A2228): https://www.sigmaaldrich.com/US/en/product/sigma/a2228<br>total SAP97 (clone RPI 197.4, ADI-VAM-PS005): https://www.enzolifesciences.com/ADI-VAM-PS005/sap97-monoclonal-antibody-rpi-197.4/<br>phospho-MARCKS (sc-12971-R): https://datasheets.scbt.com/sc-12971.pdf<br>total MARCKS (sc-6454): https://www.scbt.com/p/marcks-antibody-n-19<br>GAPDH (2118):https://www.cellsignal.com/products/primary-antibodies/gapdh-14c10-rabbit-mab/2118<br>vinculin (4650): https://www.cellsignal.com/products/primary-antibodies/vinculin-antibody/4650<br>phospho-(Ser) PKC substrate (2261S): https://www.cellsignal.com/products/primary-antibodies/phospho-ser-pkc-substrate-antibody/2261<br>phospho-ERK1/2 (9101):https://www.cellsignal.com/products/primary-antibodies/phospho-p44-42-mapk-erk1-2-thr202-tyr204-antibody/9101<br>total ERK1/2 (9102):https://www.cellsignal.com/products/primary-antibodies/phospho-p44-42-mapk-erk1-2-thr202-tyr204-antibody/9102<br>pSAP97 (T656): O'Neill AK, et al. Protein kinase Calpha promotes cell migration through a PDZ-dependent interaction with its novel substrate discs large homolog 1 (DLG1). J Biol Chem 286, 43559-43568 (2011).

Animals and other organisms

Policy information about studies involving animals; ARRIVE guidelines recommended for reporting animal research

Laboratory animals	C57BL/6NTac-PKCalpha WT mice: 3 months (4 males, 8 females), 6 months (8 males, 8 females), and 12 months old (7 males, 12 females).<br>C57BL/6NTac-PKCalpha M489V mice:3 months (6 males, 6 females), 6 months (8 males, 8 females), and 12 months old (12 males, 11 females).<br>B6;SJL-PKCalpha WT mice: 4.5 months (12 males, 12 females), 6 months (15 males, 16 females), and 12 months old (10 males, 10 females).<br>B6;SJL-PKCalpha M489V mice: 4.5 months (11 males, 12 females), 6 months (12 males, 16 females), and 12 months old (10 males, 9 females).<br>B6;SJL- Tg(APPSWE) -PKCalpha WT mice (WT tg-AD): 4.5 months (11 males, 11 females), 6 months (10 males, 11 females), and 12 months old (9 males, 9 females).<br>B6;SJL- Tg(APPSWE) -PKCalpha M489V mice (M489V tg-AD): 4.5 months (12 males, 12 females), 6 months (8 males, 6 females), and 12 months old (5 males, 14 females).
Wild animals	No wild animals were used in this study
Field-collected samples	No field-collected samples were used in this study
Ethics oversight	All animal procedures were performed with the approval of The Scripps Research Institute's Institutional Animal Care and Usage Committee (IACUC) and the University of California San Diego IACUC, and met the guidelines of the National Institute of Health detailed in the Guide for the Care and Use of Laboratory Animals. The protocol numbers approved for these procedures are 09-0004, S06288, and S11286.

Note that full information on the approval of the study protocol must also be provided in the manuscript.
